# Reporting practices of baseline and surgical variables in spinal cavernous malformation surgery: a systematic review

**DOI:** 10.1007/s10143-026-04144-w

**Published:** 2026-02-21

**Authors:** Tomas Ferreira, Louis Naraine, Joecelyn Kirani Tan, Hussein T. Malik, Mario K. Teo

**Affiliations:** 1 St Thomas’ Hospital, Westminster Bridge Road, London, UK; 2 Southmead Hospital, Bristol, UK; 3 Faculty of Biology, Medicine and Health, University of Manchester, Manchester, UK; 4 Bristol Medical School, University of Bristol, Bristol, UK; 5 Department of Neurosurgery, Southmead Hospital, Bristol, UK

**Keywords:** Spinal cavernous malformation, Intramedullary cavernous malformations, Intramedullary cavernoma, Cavernous malformations, Spinal cord cavernous malformations, Cavernoma, Baseline variables, Common data elements

## Abstract

To evaluate the consistency and completeness of baseline and surgical variable reporting in studies of spinal cord cavernous malformation (SCM) surgery, and to identify temporal and geographic trends in reporting practices. A systematic review was conducted in accordance with PRISMA guidelines (PROSPERO registration: CRD42025638978). PubMed and Embase were searched for English-language studies reporting surgical management of SCMs. Eligible studies were required to include ≥ 10 patients (prospective) or ≥ 20 patients (retrospective) and to report at least one baseline variable. Data on demographics, clinical presentation, radiological features, surgical details, and intraoperative adjuncts were extracted in duplicate and analysed descriptively. Twenty-five studies encompassing 1,633 patients met inclusion criteria. Most were single-centre (84%) with a mean sample size of 65.3 (range 20–279). All studies reported age, sex, and lesion location, whereas ethnicity (8%), comorbidities (0%), and body mass index (4%) were rarely documented. Functional grading was reported in 88% of studies, while haemorrhage (64%), lesion size (64%), and MRI sequence details (40%) were inconsistently captured. Neuromonitoring was the most frequently described intraoperative adjunct (68%), increasing from 25% of studies in the 2000s to 75% after 2010. Most publications originated from Asia (52%), followed by the Americas (28%) and Europe (20%). To our knowledge, this is the first review to systematically quantify the completeness of baseline and surgical variable reporting in SCM surgery and to map temporal and geographic patterns in those reporting practices. This review reveals pronounced heterogeneity in the reporting of variables in SCM surgery. The findings support the case for the development of consensus-derived reporting standards to improve data comparability, enable multicentre collaboration, and strengthen the evidence base for surgical management of this rare condition.

**Supplementary Information:**

The online version contains supplementary material available at 10.1007/s10143-026-04144-w.

## Introduction

Spinal cord cavernous malformations (SCMs) are rare intramedullary vascular lesions characterised by abnormally dilated, thin-walled capillaries. These malformations account for a small minority of central nervous system cavernous malformations and a modest proportion of spinal vascular pathology. Most cavernous malformations are intracranial; only approximately 5% arise in the spinal cord, and SCMs comprise roughly 5–12% of spinal cord vascular malformations [1–5]. Patients typically present with stepwise or progressive myelopathy, often related to haemorrhage, and a sizeable minority have multiple lesions or an associated intracranial cavernous malformation [6, 7].

Surgery is widely adopted for symptomatic SCMs, with contemporary series and meta-analyses suggesting better long-term neurological outcomes after resection than with conservative care, particularly when gross-total resection is achieved and surgery is undertaken early after symptom onset [1, 2, 8]. Natural-history studies report an annual haemorrhage rate of roughly 2–9%, depending on cohort composition and definition of haemorrhage, with prior bleeding emerging as the strongest predictor of subsequent events [8–12].

Despite this maturing evidence base, the SCM literature remains methodologically heterogeneous. Studies vary in design, cohort size, and follow-up. There is also wide variation in what is reported at baseline, including demographics, preoperative neurological status, haemorrhage history, imaging characteristics such as lesion level and size, and key surgical details or adjuncts [2, 7, 13]. Such inconsistency limits comparability across centres, complicates meta-analysis and prognostic modelling, and impedes the design of multicentre registries and trials [7, 13]. These challenges mirror broader concerns addressed by reporting frameworks for observational research, which advocate transparent and standardised variable definition and disclosure [14, 15]. Similar concerns regarding heterogeneity of baseline reporting have recently been documented in pituitary adenoma surgery, underscoring the wider relevance of harmonised reporting practices across neurosurgical subspecialties [16].

Here, we seek to systematically audit the consistency of reporting demographic, clinical, radiological, and surgical variables in the SCM surgical literature and to evaluate temporal and geographic trends in reporting practices in the modern era. By identifying gaps and inconsistencies, this review seeks to inform the development of standardised reporting frameworks, improving the quality and harmonisation of reporting in SCM research. Establishing consistent reporting practices could serve as the foundation for future collaborative registries and common data elements, ultimately improving both clinical care and the quality of surgical evidence.

## Materials and methods

### Protocol and registration

This protocol was registered with PROSPERO (CRD42025638978) and conducted in accordance with the Preferred Reporting Items for Systematic Reviews and Meta-Analyses (PRISMA) guidelines [17].

## Search strategy

A systematic search of PubMed and Embase was performed to identify all studies reporting surgical management of spinal cord cavernous malformations (SCMs). The core search string combined the terms *spinal cavernoma surgery*, *spinal cavernous malformation surgery*, and *intramedullary cavernoma surgery*. Additional searches included combinations of *spinal cord cavernous malformations*, *spinal cavernomas*, *intramedullary cavernomas*, *intramedullary cavernous malformations*, and the terms *spine* with *cavernous* or *cavernoma* restricted to title/abstract fields. Searches were limited to publications in English between 1995 and 2025. The full search string is provided in Supplementary Appendix 1.

## Eligibility criteria

Inclusion criteria:

- Full-text primary studies, English language, published 1995–2025.
- Human patients with spinal cord cavernous malformations.
- Surgical management reported.
- At least one baseline characteristic reported such as demographic variables, neurological status, or imaging findings.
- Prospective studies with ≥ 10 patients or retrospective studies with ≥ 20 patients.
- All randomised controlled trials.

Exclusion criteria:

- Case reports or small case series below the thresholds above.
- Reviews, meta-analyses, editorials, letters, conference abstracts.
- Mixed pathology cohorts without separately extractable SCM data.
- Studies without extractable baseline characteristics.
- Non-human or in vitro studies.
- Non-English publications.

These minimum sample-size thresholds were chosen a priori to reduce instability from very small case series, which are common in rare diseases and often demonstrate inconsistent or incomplete reporting.

## Study selection

Three reviewers (LN, JKT, HM) independently screened titles and abstracts in a blinded manner. Full texts were subsequently reviewed independently in triplicate to confirm eligibility. Discrepancies were resolved through discussion, with arbitration by a fourth reviewer (TF).

## Data extraction

Data were extracted from full-text articles by the authors (TF, LN, JKT, HM) using a predefined proforma in Microsoft Excel (Microsoft Inc., Seattle, WA). Extracted variables included: (1) study details (first author, year of publication, journal, study location); (2) study design (prospective/retrospective design, sample size); patient demographics (age, sex, ethnicity, comorbidities, family history); (3) clinical presentation variables (symptom duration, neurological status (e.g., ASIA score, sensory deficits), functional impairment); (4) radiological variables (lesion location (spinal level), lesion size/volume, presence of haemorrhage); and (5) surgical details (approach, use of intraoperative adjuncts (e.g., neuromonitoring, navigation)). The first author (TF) verified the extracted data for every 5th paper included to ensure internal validity. Baseline data for each study were collected and a complete list of these can be found in the Supplementary Materials.

### Data analysis

Descriptive statistics were generated using Microsoft Excel. Frequencies of reporting were calculated for each baseline variable. Trends in reporting were examined across three time periods (1990s, 2000 s, and 2010–2023) and by geographic region. The primary outcome was the proportion of studies reporting demographic, clinical, and radiological variables. Secondary outcomes included temporal and geographic trends in reporting practices, with emphasis on identifying inconsistencies and gaps in the literature.

## Risk of bias assessment

Formal risk of bias assessment was not undertaken, as the aim of this review was to evaluate reporting practices rather than to pool clinical outcomes.

## Results

### Study characteristics

**Fig. 1:**
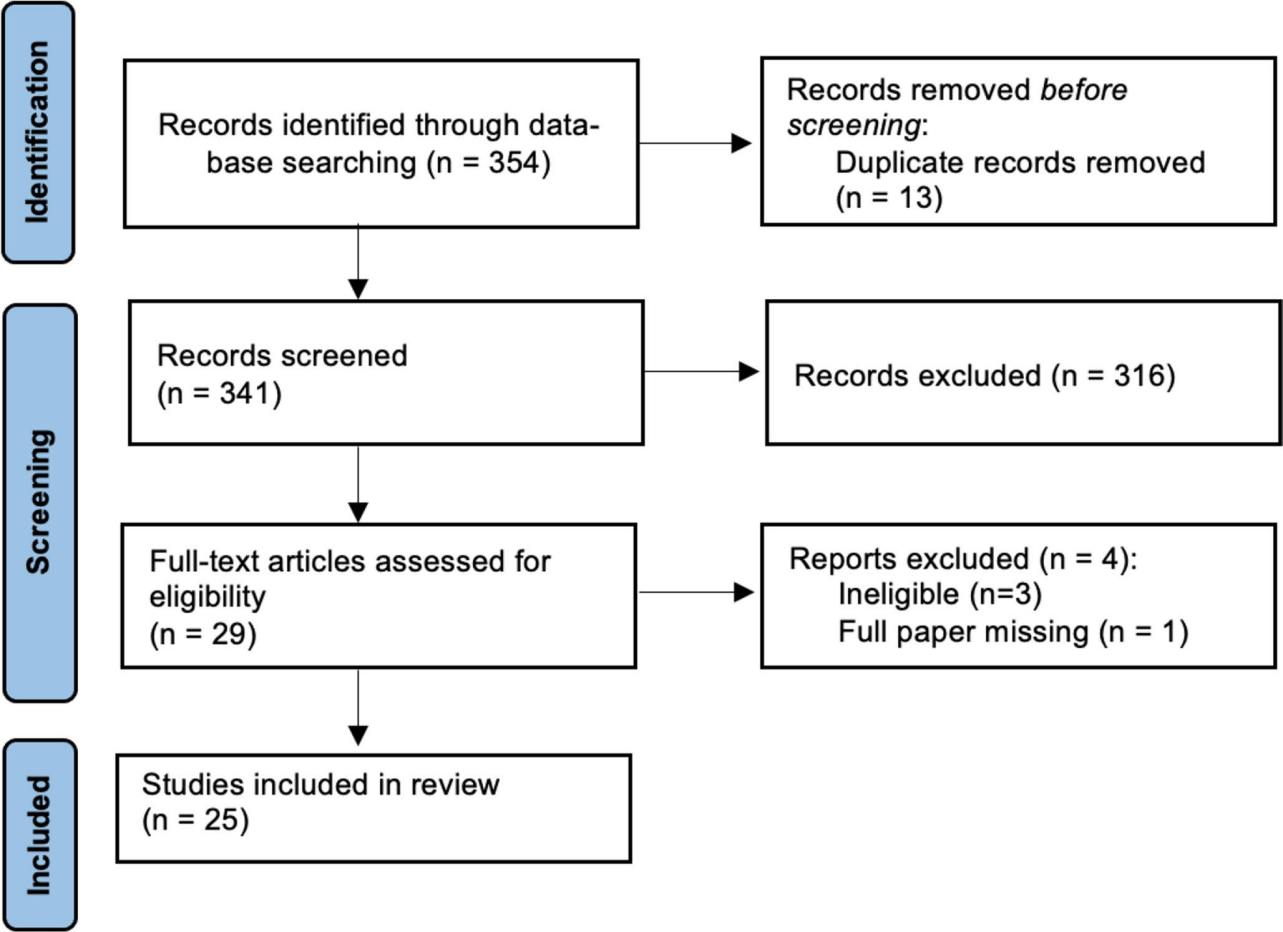
PRISMA flow diagram illustrating study selection process

Twenty-five studies published between 1995 and 2025 met inclusion criteria, encompassing a total of 1,633 surgically treated patients (Supplementary Table 1) [37–60]. The mean year of publication was 2016.4 (range 2006–2025). The majority were single-centre series (21/25, 84.0%), with only four (16.0%) conducted across multiple institutions. The mean surgical sample size was 65.3 patients (range 20–279). The study selection process is summarised in the PRISMA flow diagram (Fig. 1).

### Patient demographics

**Fig. 2:**
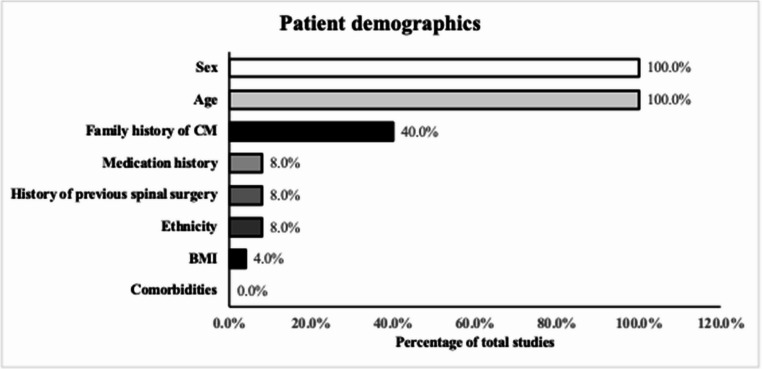
Proportion of included studies reporting demographic variables

Reporting of demographic characteristics was variable (Fig. 2). All studies documented patient age (25/25, 100.0%) and sex (25/25, 100.0%). Ethnicity was rarely reported (2/25, 8.0%), and only one study (4.0%) mentioned body mass index. Comorbidity data (0/25, 0.0%), medication history (2/25, 8.0%), previous spinal surgery (2/25, 8.0%), and family history of cavernous malformation (10/25, 40.0%) were infrequently included. In summary, demographic reporting was largely limited to age and sex, with other clinically relevant variables seldom documented.

### Clinical presentation

**Fig. 3:**
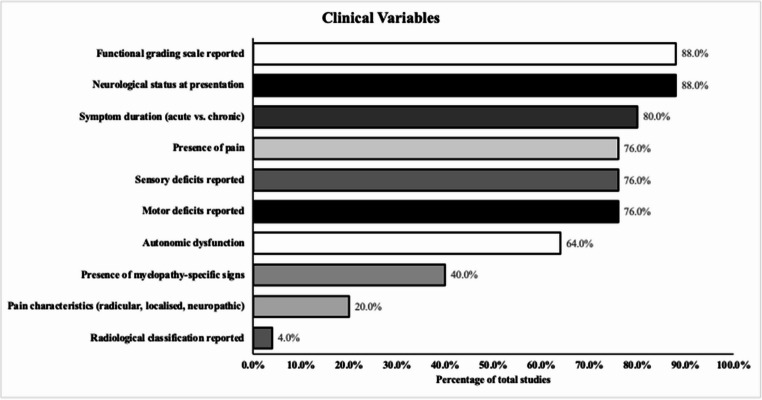
Proportion of included studies reporting clinical variables

Most studies reported neurological status at presentation, including motor (19/25, 76.0%), sensory (19/25, 76.0%), and autonomic dysfunction (16/25, 64.0%) (Fig. 3). Pain characteristics were described in 5/25 (20.0%). Symptom duration (acute vs. chronic) was reported in 20/25 (80.0%). Myelopathy-specific signs were recorded in 10/25 (40.0%). Functional grading scales (e.g., ASIA, McCormick) were used in 22/25 (88.0%), and overall neurological status was documented in 22/25 (88.0%). Thus, although neurological status at presentation was usually described, other elements such as pain characteristics and myelopathy-specific signs were inconsistently reported.

### Radiological variables

Radiological reporting beyond lesion level and basic imaging modality was highly variable, with important descriptors such as haemorrhage status, lesion size, and MRI sequences frequently omitted. Lesion location (spinal level) was universally reported (25/25, 100.0%), and imaging modality was specified in 24/25 (96.0%). Presence of haemorrhage (16/25, 64.0%) and lesion size (16/25, 64.0%) were variably documented. MRI sequences were detailed in 10/25 (40.0%). Only six studies (24.0%) reported multiple haemorrhages, three (12.0%) described oedema or myelomalacia, and three (12.0%) reported mass effect or cord compression.

### Surgical variables and intraoperative adjuncts

Surgical approach was incompletely reported, with seven studies (28.0%) not specifying the approach used. Among the 18 studies that did report an approach, the posterior route was most common (18/18, 100%), with occasional use of lateral (3/18, 16.7%) and anterior approaches (2/18, 11.1%).

Extent of resection (EOR) was reported in 18 of 25 studies (72.0%). Among these 18 reporting studies, gross-total resection was described in all 18 (18/18, 100.0%), while subtotal resection was also described in 13 (13/18, 72.2%). The remaining seven studies (7/25, 28.0%) did not provide any EOR information. Overall, reporting of surgical approach and EOR varied substantially across studies.

Neuromonitoring was the most frequently described adjunct (17/25, 68.0%), followed by intraoperative ultrasound (7/25, 28.0%). Neuronavigation was reported in only one study (4.0%), and fluorescein dye or other contrast techniques in one study (4.0%). Seven studies (28.0%) did not report use of any intraoperative adjuncts. Adjunct reporting increased over time, with neuromonitoring documented in 25.0% of studies in the 2000 s, 76.9% in the 2010 s, and 75.0% in the 2020s. Table 1 summarises the frequency of reporting of clinical, radiological, and surgical variables in included studies. Adjunct use showed considerable heterogeneity, with neuromonitoring the only technique reported consistently enough to identify temporal trends.

**Table 1:** Frequency of reporting of clinical, radiological, and surgical variables in studies of spinal cord cavernous malformation surgery

Domain	Variable	*n*	%
*Preoperative imaging reported*			
	Lesion location specified (spinal level)	25	100.00%
	Imaging modality specified (MRI/CT)	24	96.00%
	Presence of haemorrhage	16	64.00%
	Lesion size reported	16	64.00%
	MRI sequences specified (T1, T2, GRE, SWI)	10	40.00%
	Multiple haemorrhages (≥ 2 preop events)	6	24.00%
	Oedema/myelomalacia	3	12.00%
	Mass effect/spinal cord compression	3	12.00%
*Surgical approach*			
	Posterior (laminectomy/laminoplasty)	18	72.00%
	Lateral	3	12.00%
	Anterior (corpectomy/ACDF)	2	8.00%
	Not reported	7	28.00%
*Extent of resection*			
	Extent of resection reported	18	72.00%
	Gross total*	18	100.00%
	Subtotal*	13	72.22%
	Not reported*	7	28.00%
*Use of intraoperative adjuncts*			
	Neuromonitoring	17	68.00%
	Neuronavigation	1	4.00%
	Intraoperative ultrasound	7	28.00%
	Fluorescein dye/contrast	1	4.00%
	None reported	7	28.00%
*Reporting quality metrics*			
	Follow-up duration reported	24	96.00%
	Loss to follow-up mentioned	6	24.00%
	None reported	1	4.00%

*Percentages for gross-total and subtotal resection are calculated among studies that reported extent-of-resection data (*n* = 18)

### Reporting quality metrics

Follow-up duration was reported in 24/25 (96.0%), but loss to follow-up was explicitly mentioned in only six (24.0%). One study (4.0%) did not provide any follow-up information. Despite near-universal documentation of follow-up duration, explicit reporting of attrition remained uncommon.

### Temporal and geographic trends

Despite increasing publication volume and modest improvements in specific variables, wide heterogeneity in reporting persisted across eras and regions. Most studies originated from Asia (13/25, 52.0%), followed by the Americas (7/25, 28.0%) and Europe (5/25, 20.0%). Within individual countries, China accounted for the largest number of publications (11/25, 44.0%), with the United States contributing seven (28.0%), and smaller numbers from France, Japan, Switzerland, Germany, and South Korea. The majority of series were single-centre (21/25, 84.0%), with only four multicentre studies (16.0%), underscoring the predominance of institution-specific reporting.

Over time, the number of published series increased markedly after 2006, with the greatest output during the 2010 s (13/25, 52.0%). Eight studies (32.0%) were published in the 2020 s to date, compared with only four (16.0%) in the 2000s. The quality of reporting improved modestly over time: for example, use of intraoperative neuromonitoring was documented in just 25.0% of studies published in the 2000 s, rising to 76.9% in the 2010 s and 75.0% in the 2020s. In contrast, other adjuncts such as ultrasound and neuronavigation were inconsistently reported throughout the study period, and fluorescein use was rare. Despite increased volume and improved documentation of certain variables, wide heterogeneity in demographic, clinical, radiological, and surgical reporting persisted across decades.

## Discussion

This systematic review demonstrates substantial variability in reporting practices across the surgical literature on SCMs. Although fundamental variables such as age, sex, lesion level, imaging modality, and functional grading were consistently described, there was marked inconsistency in the documentation of other clinically relevant details. Key gaps included comorbidities, ethnicity, body mass index, medication and family history, prior spinal surgery, and radiological descriptors such as oedema, mass effect, multiple haemorrhages, and MRI sequences. Reporting of intraoperative adjuncts and the extent of resection was equally heterogeneous, and the quality of follow-up reporting varied widely. These findings highlight the need for harmonised reporting standards to improve the reproducibility and interpretability of SCM research.

### Findings in the context of the literature

Efforts to standardise clinical research reporting have gained momentum across medicine, driven by the recognition that inconsistent data capture impedes comparability, synthesis, and reuse of information. Common Data Elements (CDEs) provide structured, consensus-derived definitions that facilitate uniform data collection across studies [18]. Developed through multidisciplinary collaboration, CDE frameworks are now widely used in clinical trials and observational research to ensure that datasets are interoperable and comparable across centres [19–21]. The National Institute of Neurological Disorders and Stroke (NINDS) launched its CDE initiative in 2006, establishing standards for neurological disorders such as epilepsy, Parkinson’s, traumatic brain injury, Chiari malformations, and stroke [22–25]. These frameworks have accelerated discovery, reduced duplication, and allowed researchers in resource-limited settings to contribute to high-quality multicentre studies through harmonised data collection [26].

Variability in baseline and operative reporting has been documented in other neurosurgical subspecialties, notably in pituitary adenoma surgery [16], where heterogeneity in demographic, endocrine, and ophthalmic data was shown to impede synthesis and benchmarking. Our findings confirm that this challenge extends to SCMs. In a rare disease with small case series and limited prospective data, heterogeneity carries disproportionate consequences: meta-analyses are weakened, prognostic modelling becomes unreliable, and the evidence base guiding surgical decision-making remains fragmented.

This inconsistency extends even to clinically relevant variables with known pathophysiological significance. Cerebral cavernous malformations, which share overlapping biological and genetic mechanisms with SCMs, occur in approximately 0.4–0.9% of the general population [27, 28]. Around 80% of CCMs are sporadic, typically presenting as solitary lesions often associated with a developmental venous anomaly, whereas ~ 20% represent familial or syndromic forms arising from autosomal-dominant pathogenic variants in KRIT1, CCM2, or PDCD10 [27, 29–35]. Familial cavernomatosis frequently exhibits variable penetrance, and spinal involvement is well documented, with up to 70% of individuals carrying KRIT1 variants demonstrating at least one spinal lesion [36]. Yet, family history was reported in only 40% of SCM surgical studies, and explicit genetic or syndromic screening was almost never mentioned. This omission represents a missed opportunity to identify hereditary clustering, stratify risk, and harmonise data collection. Incorporating genetic and family history variables into future common data element frameworks could therefore strengthen both the internal validity and translational relevance of SCM research.

### Clinical and research implications

Incomplete or inconsistent reporting limits the ability to identify predictors of outcome, define optimal timing for surgery, or evaluate the efficacy of intraoperative adjuncts such as neuromonitoring or ultrasound. Without uniform baseline descriptors, comparing postoperative recovery or rehaemorrhage risk across studies becomes speculative. Standardisation is therefore not an academic exercise but a prerequisite for accurate prognostication, patient counselling, and evidence-based practice.

Developing a consensus-driven CDE for SCM research would address these deficiencies. At a minimum, such a framework should include: (1) demographic information (age, sex, ethnicity, comorbidities, family history); (2) clinical presentation and neurological status using a validated scale; (3) radiological features (spinal level, size, haemorrhage status, MRI sequences); (4) surgical details (approach, adjuncts, extent of resection); and (5) follow-up parameters (duration, attrition, recurrence). Consistent adoption of such elements would enhance data comparability, reduce research waste, and enable future registries and multicentre collaborations to generate high-fidelity datasets capable of informing practice guidelines.

### Temporal and geographic trends

Reporting quality showed modest improvement over time, particularly in the documentation of intraoperative neuromonitoring, which increased from 25% of studies in the 2000 s to more than 75% in the 2010 s and 2020s. Nevertheless, key variables remained inconsistently captured even in recent publications. Most included studies originated from Asia, with fewer contributions from Europe and the Americas, and only four were multicentre. This geographic skew highlights the need for broader international collaboration and uniform data standards to ensure representativeness and reproducibility across health-care systems.

### Limitations

This review has several limitations. First, only two databases (PubMed and Embase) were searched, and studies not indexed in these sources or published in languages other than English may have been missed. Second, inclusion thresholds for sample size may have excluded smaller series, which are not uncommon given the rarity of spinal cavernous malformations. Third, variability in study designs across the included literature may affect the generalisability of the findings. Finally, the analysis was descriptive and cannot establish causal associations; observed temporal and geographic trends should therefore be interpreted with caution. These limitations notwithstanding, the review provides a comprehensive overview of reporting practices in the surgical literature on spinal cavernous malformations and highlights areas where greater standardisation is required.

## Conclusion

This systematic review provides the first comprehensive assessment of reporting practices in SCM surgery over the past three decades. Given the rarity of SCMs and the already relatively limited number of neurosurgical case series, consistent and complete reporting of baseline characteristics is essential to enable meaningful comparison across studies and reliable synthesis of outcomes. The findings of this review highlight substantial heterogeneity in the reporting of demographic, clinical, radiological, and surgical variables, underscoring the need for greater methodological uniformity. Establishing consensus-derived reporting standards will be critical to improving the quality, comparability, and translational value of future research. This review represents a first step towards harmonisation of SCM data reporting. Future multicentre registries adopting standardised datasets will be essential to clarify prognostic factors, guide surgical decision-making, and optimise outcomes in this rare but clinically important condition.

## Supplementary Information

Below is the link to the electronic supplementary material.

Supplementary Material 1

Supplementary Material 2

Supplementary Material 3

## Data Availability

No datasets were generated or analysed during the current study.
