# Supplementary material for: Reporting practices of baseline and surgical variables in spinal cavernous malformation surgery: a systematic review
Source: Neurosurg Rev. 2026 Feb 21;49(1):239. doi: 10.1007/s10143-026-04144-w (PMC12923459; doi:10.1007/s10143-026-04144-w)
Supplement: Supplementary file 1 — Supplementary Material 1 [file 10143_2026_4144_MOESM1_ESM.docx]

**Data items**

1. Study details
   1. First author
   2. Year of publication
   3. Journal
   4. Location of study
2. Study design
   1. Study design (prospective, retrospective, RCT)
   2. Study period
   3. Sample size
3. Patient Demographics
   1. Age (mean/median)
   2. Sex distribution
   3. Ethnicity distribution
   4. Comorbidities (if reported)
   5. Body mass index
   6. History of previous spinal surgery
   7. Family history of cavernous malformations
4. Clinical Presentation Variables
   1. Symptom duration (acute vs chronic)
   2. Neurological status at presentation (e.g., ASIA score, McCormick grade)
   3. Presenting symptoms (motor, sensory, pain, autonomic dysfunction)
5. Preoperative imaging
   1. Imaging modality used (MRI, CT)
   2. Lesion location (spinal level)
   3. Lesion size (if reported)
   4. Presence of haemorrhage
6. Surgical Variables
   1. Surgical approach (posterior, anterior, minimally invasive)
   2. Use of intraoperative adjuncts (neuromonitoring, navigation)
