# Supplementary material for: Reporting practices of baseline and surgical variables in spinal cavernous malformation surgery: a systematic review
Source: Neurosurg Rev. 2026 Feb 21;49(1):239. doi: 10.1007/s10143-026-04144-w (PMC12923459; doi:10.1007/s10143-026-04144-w)
Supplement: Supplementary file 2 — Supplementary Material 2 [file 10143_2026_4144_MOESM2_ESM.docx]

Search string: spinal cord cavernous malformations[Title/Abstract] OR spinal cavernous malformations[Title/Abstract] OR spinal cavernomas[Title/Abstract] OR intramedullary cavernomas[Title/Abstract] OR intramedullary cavernous malformations[Title/Abstract] OR (spine[Title/Abstract] AND cavernous[Title/Abstract]) OR (spine[Title/Abstract] AND cavernoma[Title/Abstract])
