# Supplementary material for: Reporting practices of baseline and surgical variables in spinal cavernous malformation surgery: a systematic review
Source: Neurosurg Rev. 2026 Feb 21;49(1):239. doi: 10.1007/s10143-026-04144-w (PMC12923459; doi:10.1007/s10143-026-04144-w)
Supplement: Supplementary file 3 — Supplementary Material 3 [file 10143_2026_4144_MOESM3_ESM.docx]

|  | **Authors** | **Study Title** | **Country** | **Surgical management sample size** | **Total sample size management sample size** |
| --- | --- | --- | --- | --- | --- |
| 1 | Cohen-Gadol et al. 2006 [25] | Coexistence of intracranial and spinal cavernous malformations: a study of prevalence and natural history | USA | 20 | 25 |
| 2 | Jallo et al. 2006 [21] | Clinical presentation and optimal management for intramedullary cavernous malformations | USA | 160 | 160 |
| 3 | Kim et al. 2006 [20] | Analysis of pain resolution after surgical resection of intramedullary spinal cord cavernous malformations | USA | 23 | 53 |
| 4 | Labauge et al. 2008 [34] | Outcome in 53 patients with spinal cord cavernomas | France | 26 | 26 |
| 5 | Lu et al. 2010 [22] | Clinical presentation and surgical management of intramedullary spinal cord cavernous malformations | USA | 22 | 22 |
| 6 | Choi et al. 2011 [39] | The clinical features and surgical outcomes of patients with intramedullary spinal cord cavernous malformations | South Korea | 20 | 20 |
| 7 | Liang et al. 2011 [33] | Management and prognosis of symptomatic patients with intramedullary spinal cord cavernoma | China | 32 | 107 |
| 8 | Mitha et al. 2011 [35] | Outcomes following resection of intramedullary spinal cord cavernous malformations: a 25-year experience | USA | 25 | 67 |
| 9 | Tong et al. 2012 [23] | Clinical presentation and surgical outcome of intramedullary spinal cord cavernous malformations | China | 32 | 40 |
| 10 | Ardeshiri et al. 2016 [18] | A retrospective and consecutive analysis of the epidemiology and management of spinal cavernomas over the last 20 years in a single center | Germany | 58 | 85 |
| 11 | Zhang et al. 2016 [27] | Comparison of outcome between surgical and conservative management of symptomatic spinal cord cavernous malformations | China | 54 | 54 |
| 12 | Azad et al. 2018 [31] | Long-term effectiveness of gross-total resection for symptomatic spinal cord cavernous malformations | USA | 35 | 35 |
| 13 | Velz et al. 2018 [40] | The current management of spinal cord cavernoma | Switzerland | 85 | 85 |
| 14 | Goyal et al. 2019 [24] | Clinical presentation, natural history and outcomes of intramedullary spinal cord cavernous malformations | USA | 32 | 32 |
| 15 | Li et al. 2019 [28] | Differences in the electrophysiological monitoring results of spinal cord arteriovenous and intramedullary spinal cord cavernous malformations | China | 111 | 111 |
| 16 | Ren et al. 2019 [26] | Coexistence of intracranial and spinal cord cavernous malformations predict aggressive clinical presentation | China | 81 | 96 |
| 17 | Ren et al. 2019 [36] | Surgical approaches and long-term outcomes of intramedullary spinal cord cavernous malformations: a single-center consecutive series of 219 patients | China | 40 | 53 |
| 18 | Zhang et al. 2021 [32] | Long-term surgical outcomes and prognostic factors of adult symptomatic spinal cord cavernous malformations | China | 80 | 80 |
| 19 | Cai et al. 2023 [8] | Surgical outcomes of symptomatic intramedullary spinal cord cavernous malformations: analysis of consecutive cases in a single center | China | 214 | 219 |
| 20 | Kurokawa et al. 2023 [19] | Acceptance of Early Surgery for Treatment of Spinal Cord Cavernous Malformation in Contemporary Japan | Japan | 98 | 98 |
| 21 | Liao et al. 2023 [37] | Surgical outcomes of spinal cavernous malformations: A retrospective study of 98 patients | China | 29 | 29 |
| 22 | Rauschenbach et al. 2023 [29] | Functional neurological outcome of spinal cavernous malformation surgery | Germany | 279 | 279 |
| 23 | Li et al. 2024 [30] | Intraoperative changes in electrophysiological monitoring can be used to predict clinical outcomes in patients with spinal cavernous malformation | China | 21 | 21 |
| 24 | Tian et al. 2024 [38] | Surgical timing and long-term outcomes in patients with severe haemorrhagic spinal cord cavernous malformations | China | 21 | 29 |
| 25 | Früh et al. 2025 [41] | Treatment of spinal cavernous malformations: A single-center case series | Germany | 35 | 52 |

**Supplementary Table 1** - Characteristics of included studies on surgical management of spinal cord cavernous malformation
